# Availability and readiness of diabetes health facilities to manage tuberculosis in Tanzania: a path towards integrating tuberculosis-diabetes services in a high burden setting?

**DOI:** 10.1186/s12889-019-7441-6

**Published:** 2019-08-14

**Authors:** Festo K. Shayo, Sigfrid Casmir Shayo

**Affiliations:** 1 Department of Internal Medicine, Muhimbili University of Health and Allied Sciences, P. O Box 14087, Dar es Salaam, Tanzania; 2 Department of Global Health Entrepreneurship, Division of Public Health, Graduate School of Tokyo Medical and Dental University, Tokyo, Japan; 3 Department of Diabetes and Endocrine Medicine, Kagoshima University, Kagoshima, Japan

**Keywords:** Availability, Readiness, Diabetes facilities, Tuberculosis, Services, Tanzania

## Abstract

**Background:**

The burden of tuberculosis (TB) and diabetes mellitus (DM) is rising and substantially affecting the low-income countries, including Tanzania. Integrated management of TB and DM is becoming of importance in TB high burden countries. In this study, we sought to assess the availability and readiness of diabetes facilities to manage TB in Tanzania.

**Methods:**

The present study was based on a secondary analysis of the 2014–2015 Tanzania Service Provision Assessment Survey data. We calculated the service availability as a percentage of diabetes facilities offering TB services: diagnosis and treatment. Regarding the readiness of diabetes facilities to provide TB management, we calculated based on the three domains: staff training and guideline, diagnostics, and medicines as identified by World Health Organization-Service Availability and Readiness Assessment (SARA) manual. A score of at least half (≥50%) of the indicators listed in each of the three domains was considered as high readiness. We used a descriptive statistics to present our findings.

**Results:**

There were 619 DM facilities all over the country of which only 238 (38.4%) had TB services.72.6 and 62.6% of these DM facilities with TB services were publicly owned and located in rural settings respectively. Generally, DM facilities had low readiness to manage TB; 12·6%. More specifically, all DM facilities had low readiness in terms of trained staff and guidelines. However, in the domain of diagnostics and medications, higher levels of care (hospitals) had a comparatively higher level of readiness to manage TB.

**Conclusion:**

Most of the DM facilities had low availability and readiness to manage TB. The findings of our study display an urgent need to mobilize important resources to enhance the integration of TB services in DM facilities. This includes medications, management guidelines, diagnostics, and health professionals who have received refresher training on TB/DM co-management. However, presently, few DM facilities may be allowed to start managing TB as per the Strategic and Action Plan for the Prevention and Control of Non-Communicable Diseases in Tanzania 2016–2020.

## Background

Despite a decline in active TB mortality since 1990, the disease is still one of the top ten causes of mortality worldwide. In the year 2015, there were 10.4 million incident TB cases worldwide [1]. However, through the “End TB Strategy,” the World Health Organization (WHO) set targets of 90 and 80% reduction in TB mortality and incidence, respectively, by 2030 [1, 2].

TB remains one of the main causes of morbidity and mortality in low- and medium-income countries (LMICs), where the prevalence of DM is also increasing [3]. For example, Tanzania is one of the top 30 countries with a high TB burden in the world [1]. The incidence rate of TB in Tanzania has increased slightly from 125/100000 population in 2015 to 129/100000 population in 2016. TB also accounts for 5.8% of all deaths in 2014 [4].

In contrast to TB, the global DM prevalence rate has been increasing from 4.7% in the 1980s to 8.5% in 2014 [5]. In 2014 about 422 million adults were living with DM globally, and in 2016 about 1.2 million deaths were directly caused by DM [6].

In Sub-Saharan Africa (SSA), as in the rest of the world, the prevalence of DM is on the rise [7]. In 2015, the International Diabetes Federation (IDF) reported that there were 14.2 million people with DM in SSA, and projected the number to increase to 34.2 million by 2040 [8, 9]. On the other hand, Tanzania is currently experiencing a marked increase in the burden of NCDs overshadowed by communicable diseases (CD) [10]. In a nationwide survey, the prevalence of DM in the working-age population was 9.1%, while the combined prevalence of DM and pre-diabetes was 20% in 2012 [11]. Moreover, DM was the 7th most common cause of death among people aged 5 years and above in 2014 [12].

DM poses a great challenge to meeting the “End TB Strategy” set targets by 2030 [13, 14]. In the year 2012, over 1 million people had TB/DM comorbidity globally [14]. Substantial evidence already shows that patients with DM have an increased risk of developing TB than the general population [15, 16]. It has also been shown that DM patients with TB tend to have poor glycemic control [16, 17].

An increase in the prevalence of TB-DM comorbidity is becoming of public health importance in Tanzania and other LMICs [18]. One case-control study involving 803 confirmed TB cases and 350 controls was conducted in Tanzania to assess the association between DM and TB. In this study, the prevalence of DM among pulmonary TB patients was 16.7% compared to 9.4% among those without TB [19]. Although a comprehensive national wide data on the burden of TB-DM comorbidity is unknown in Tanzania, the strategies to tackle the growing burden of TB/DM comorbidity are ongoing.

The WHO recommend bidirectional screening of DM and TB [20], however, this has been implemented to varying degrees in LMICs such as Tanzania [6]. Bidirectional screening for TB and DM is reported to give high yield for TB among DM patients and vice versa [21–23]. In addition, this approach not only reduces the incidence and spread of TB but also slows down the development of DM complications [21].

Responding to WHO recommendation, The Tanzanian Ministry of Health, Community Development, Gender, Elderly and Children (MoHCDGEC) developed the National guideline for TB/DM collaborative care [22]. Moreover, the Tanzanian NCDs strategic plan II (2016–2020) endeavors to train healthcare providers, select sites for phase implementation, strengthen referral and linkage mechanism and scale up TB/DM services [22]. However, the Tanzania National TB and Leprosy Programme (NTLP) does not clearly comment on how to implement the bidirectional TB-DM screening/diagnosis [23].

In Tanzania, DM service is provided at all levels of healthcare. Level 1 is primary healthcare: comprising health centers, dispensaries, and clinics (a health post that provide basic health care and family planning). According to the Tanzania national health intervention policy, the primary healthcare facilities should provide DM-related preventive services, perform routine examination and investigations, diagnose and treat DM patients including referring complicated cases to higher-level facilities [24, 25]. Level 2 is the first level hospital (district hospitals), level 3 (regional referral hospitals), level 4 (zonal referral hospitals), and level 5 (national hospitals) [26]. The services available for each of the disease’s conditions are provided within the health facility departments. For instance, the DM clinic is within the health facility and it caters for all types of DM conditions. The number of DM patients enrolled for diabetes care varies significantly across different levels of healthcare. For instance, according to DM healthcare survey 2014 report, zonal referral hospitals had enrolled more patients than the regional hospitals [27]. It is reported that the average number of enrolled patients per DM clinic in zonal hospitals was 4063 [27].

It is expected that a healthcare system to ensure access to and quality of health services, including service availability, the physical presence of facilities, and readiness capacity to deliver the services offered [28]. The WHO Collaborative Framework for Care and Control of TB and DM provides guidelines to establish mechanisms of collaboration, including joint coordination, bidirectional surveillance and screening, and guidelines for screening and management of TB-DM patients [6]. Furthermore, the WHO Service Availability and Readiness Assessment (SARA) proposed the minimum standards for health service delivery and readiness for specific health interventions, including DM and TB [28].

There is limited information on the capacity and readiness of Tanzanian healthcare facilities to manage the NCDs including DM. Recently, few studies revealed that healthcare facilities were not sufficiently ready to manage patients with DM, hypertension, and chronic respiratory diseases in Tanzania [29]. Worse still, the capacity and readiness of the few available DM facilities to manage TB are yet to be realized. Therefore, it is within this context; we sought to explore the availability and readiness of DM facilities to manage TB in Tanzania. The findings of our study will help to understand the extent to which the healthcare facilities are ready for TB-DM co-management in Tanzania.

## Methods

### Data source

The current study is based on the secondary analysis of the 2014–2015 TSPA Survey dataset which is publicly available on request from the Demographic and Health Survey Program repository: https://dhsprogram.com/data/available-datasets.cfm

### Sampling technique and sample size

**Fig. 1:**
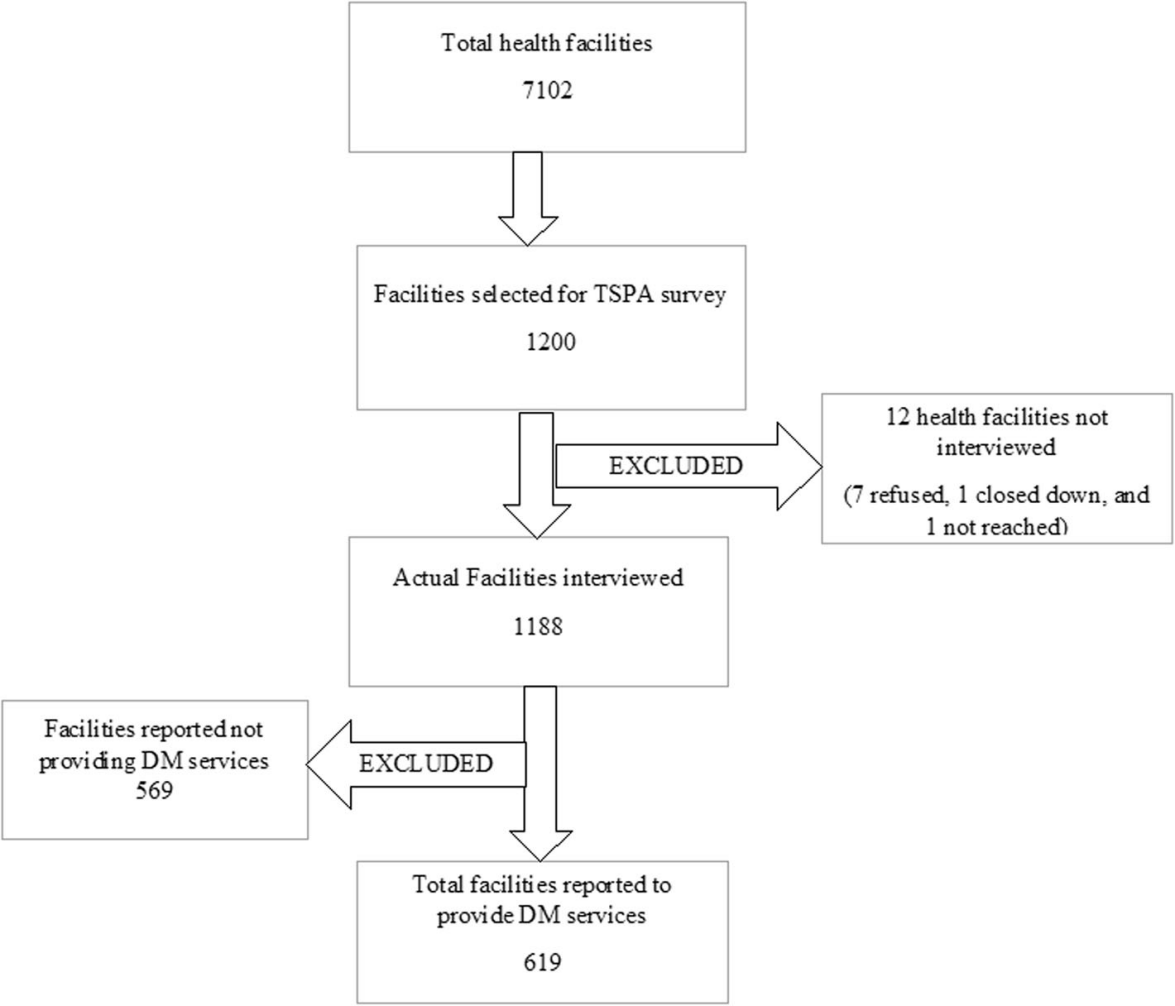
Selection of health facilities included in the current analysis

We used a systematic random sampling to select a total of 1200 health facilities for the TSPA survey. The sampling techniques and the sample size were designed to offer nationally representative findings. Of the 1200 health facilities, the following did not meet the inclusion criteria and were excluded; seven refused to participate, four were closed on the day of the survey, 569 were not providing DM services. Therefore, a total of 619 health facilities were included in the current analysis. For more details, refer to Fig. 1.

### Measures of variables

The first outcome variable was “availability” of TB services in diabetes facilities. The availability was calculated as a percentage of diabetes facilities providing both diagnosis and treatment of TB. Service availability was defined as “percentage of diabetes facilities offering TB services”. TB services means TB diagnosis and treatment.

The second outcome variable was “readiness” of the diabetes health facilities. We defined the readiness as “the capacity of the diabetes facility to provide management for TB ”. The diabetes facility was considered having “high readiness” if they scored at least a half (≥50%) and “low readiness” if they scored less than half (< 50%) of the indicators in each of the three domains suggested by WHO-SARA reference manual [30]. The first domain was staff and guidelines which had four indicators; the presence of a guideline for TB diagnosis and treatment, guidelines for TB infections control, at least one staff who received a refresher training in TB diagnosis and treatment. The facilities with guidelines were categorized as “Yes” while those without such guidelines were categorized as “No.” similarly, facilities with at least one staff member that had received refresher training in TB diagnosis and treatment within 24 months were categorized as “Yes”, otherwise were categorized as “No”. The second domain was diagnostic equipment which had one indicator as the main TB diagnostic methods in Tanzania context; the sputum smear microscopy. The facilities with sputum smear microscopy were categorized as “Yes”, otherwise were categorized as “No”. The third domain was a basic medicine which had one indicator: the availability of the first-line fixed TB-drugs combination; Isoniazid, Pyrazinamide, Rifampicin, and Ethambutol. The facilities with the four TB-drug combinations were categorized as “Yes”, otherwise were categorized as “No”.

More details regarding the presents study methodology has been published previously [29].

#### Explanatory variables

The facility type was categorized as a “hospital,” “health center,” and “clinic/dispensary”; residence was categorized as “urban” and “rural”; and managing authority was categorized as “public” and “private”.

### Data process and analysis

Data were analyzed using SPSS version 22 (SPSS, Chicago, IL). All estimates were weighted to correct for non-responses and disproportionate sampling. We used the descriptive statistics to present our findings.

## Results

Table 1 represents the baseline characteristics of diabetes facilities. Of the 619, diabetes facilities, 473 (76·4%) were dispensaries and clinics, and 416 (67·3%) were located in a rural setting. When assessed by managing authority near two-thirds 400 (64·6%) were publicly owned facilities.

**Table 1:** Distribution characteristics of facilities reported to provide DM management [*N* = 619]

	Availability of DM service *n*[%]
Facility type	Yes
Hospital	42 (6·8)
Health centers	104 (16·8)
Dispensaries and Clinics	473 (76·4)
Managing authority	
Public	400 (64·6)
Private	219 (35·4)
Residence	
Urban	203 (32·7)
Rural	416 (67·3)

Regarding availability of TB services, a total of 619 facilities reported providing DM management were assessed for TB service availability. Overall, 238 (38·4%) of all diabetes facilities offer diagnosis and treatment for TB.

Table 2 represents the percentage availability of TB services according to facility characteristics. Of the 238 diabetes facilities reported providing management for TB when assessed based on facility characteristics; 17.1% were hospitals, 72.6% were publicly owned and 62.6% were rural located.

**Table 2:** Distribution characteristics of diabetes facilities reported to provide TB services (*n* = 238)

	Availability of TB service *n*[%]
Facility type	Yes
Hospital	41 [17.1]
Health centers	83 [34.8]
Dispensaries and Clinics	114 [48.1]
Managing authority	
Public	173 [72.6]
Private	65 [27.4]
Residence	
Urban	89 [37.4]
Rural	149 [62.6]

**Fig. 2:**
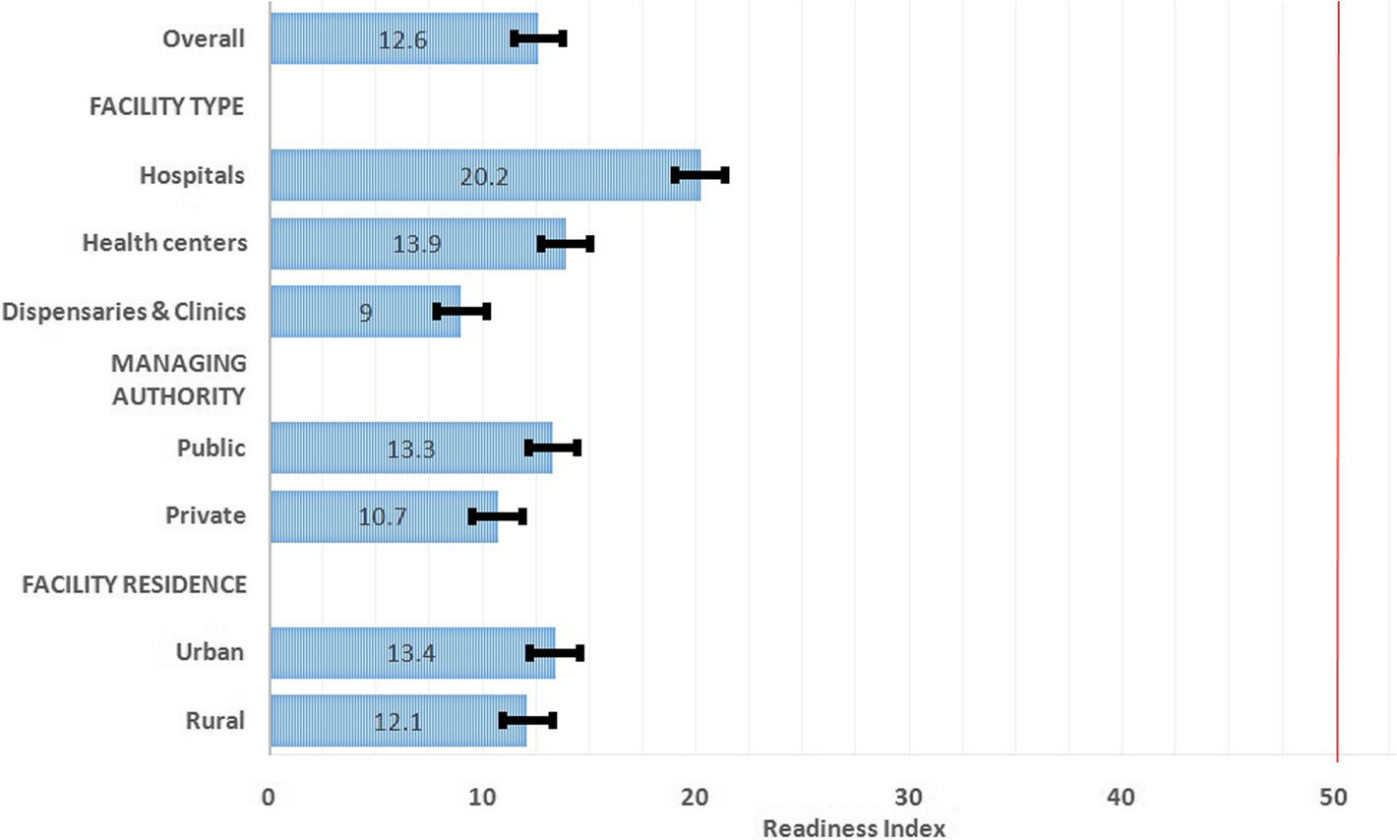
Overall readiness of diabetes facilities to provide management for TB stratified by facility characteristics (*n* = 238). The vertical red line indicates the cut off below which facility was regarded as having low readiness to manage TB. Error bar indicates standard errors

Figure 2 represents the percentage of the overall readiness of diabetes facilities to provide management for TB. The overall readiness of diabetes facilities to provide TB services was low; 12·6%. Likewise, when assessed based on facility characteristics the majority of diabetes facilities showed the low readiness to provide management for TB.

Table 3 represents the domain-specific and overall readiness of diabetes facilities to provide management for TB. None of the diabetes facilities were ready to provide management for TB in terms of trained staff and guidelines. Regarding diagnostics domain; only hospitals facility type was ready; 18·6%. On the other hand, with exception of dispensaries and clinics other facilities characteristics were ready to provide management for TB in terms medicines; hospitals 27.4%, health centers 24.3%, public facilities 22.1%, private facilities 16.8%, urban facilities 19.5%, and rural facilities 21.4%.

**Table 3:** Domain-specific readiness of DM facilities with TB services to provide management for TB stratified by facility characteristics (*N* = 238)

Variables	Facility-level			Managing authority		Facility location	
	Hospitals *n* = 41	Health centers *n* = 83	Disp. & Clin. *n* = 114	Government *n* = 173	Private *n* = 65	Urban *n* = 89	Rural *n* = 149
Staff and guidelines	*n* [index %]						
Staff Trained for TB diagnosis - Yes	1 [0·7]	2 [0·7]	5 [1.3]	7 [1·3]	1 [0·3]	1 [0·5]	6 [1·3]
Staff Trained for TB Treatment - Yes	1 [0.7]	2 [0.7]	7 [2·1]	10 [1·8]	1 [0·5]	5 [2·0]	5 [1·2]
Guideline for TB diag & treatment - Yes	37 [30.0]	70 [28.1]	60 [17.7]	128 [24·6]	38 [19.9]	61 [22.8]	107 [23.8]
Guideline for TB infection control - Yes	33 [26.6]	55 [21.9]	54 [15.9]	109 [21.0]	32 [16.5]	54 [20.2]	88 [19.7]
Mean score (±SD)	14·6 (13·8)	12·9 (12·3)	9·3 (7·6)	12·2 (10·7)	9·3 (9·0)	11·4 (10·2)	11·5 (10·4)
Diagnostics							
TB microscopy - Yes	23 [18·6]	11 [4·5]	7 [1·9]	29 [5·6]	11 [5·9]	26 [9·7]	15 [3·3]
Medicines							
First-line TB medications - Yes	34 [27·4]	60 [24·3]	54 [15·7]	115 [22·1]	33 [16·8]	52 [19·5]	96 [21·4]
Overall score (±SD)	20.2 (5.3)	13.9 (8.1)	9.0 (5.6)	13.3 (6.8)	10.7 (4.6)	13.5 (4.3)	12.1 (7.4)

## Discussion

Integrated management of TB-DM in healthcare facilities is of paramount importance especially in a setting with a high burden of TB and DM. In this study, we described the availability and readiness of Tanzanian diabetes facilities to provide TB management services.

We found that DM facilities had limited TB management services. Given the high burden of TB, DM, and TB-DM comorbidity there is a need to scale-up the availability of TB services in facilities dedicated to managing DM in Tanzania [1, 4, 12, 19, 31, 32]. Strengthening the availability of TB services in diabetes clinics may help to reduce the incidence and burden of active TB and thus enhancing the achievement “End TB Strategy” targets by 2030. We also found that public diabetes care facilities had comparatively higher availability of TB management services than private ones. This could be attributed by the fact that before 2007, TB services were mainly restricted to public health facilities [33, 34]. However, at present, we are witnessing a significant increase in the number of private health facilities providing TB management services [23]. Moreover, TB management services were available more in rural DM facilities than the urban counterpart. The fact that most healthcare facilities are located in rural settings, where more than 70% of the population live gives a reasonable explanation about this finding [35].

According to the findings of this study, DM facilities had an overall low readiness to provide TB management services. This could be due to the shortage of staff trained to co-manage TB in DM care facilities. Other reasons could be inadequate TB management guidelines, medications, and diagnostics. However, the higher levels of healthcare, public, and urban facilities with DM services had adequate medications needed to manage TB. It is important to note that all the three domains; staff and guidelines, diagnostic equipment, and medicine are altogether required for a comprehensive provision of TB services in diabetes facilities. Our study finding is comparable to three previous studies conducted in Tanzania to assess the readiness of health facilities for providing management of NCDs. Two studies were based on secondary analysis of the 2014–2015 TSPA Survey while the remaining study was a review-based paper analysis. The studies revealed that health facilities were not adequately ready to provide management of DM [36], chronic respiratory diseases [29] and hypertension [37, 38].

Bidirectional screening for TB and DM has been reported to be feasible and gives a high yield for TB among DM patients [21] as well as for DM among TB patients [21, 39]. Also, active screening and diagnosis for both diseases can reduce TB transmission and incidence and the development of DM complications [21]. Nevertheless, WHO recommends joint coordination in bidirectional surveillance, screening/diagnosis, and management of TB-DM patients [6]. In Tanzania, the Tanzania Ministry of Health has developed the guideline for TB/DM collaborative care [22]. Also, among the priority actions of the Tanzania NCDs strategic plan II (2016–2020) is to train healthcare providers on the collaborative TB-DM care, select sites for phase implementation, strengthen referral and linkage mechanisms, and scale up TB/DM services [22]. Therefore, given the high burden of TB and DM in Tanzania and our current study findings of low readiness of diabetes facilities to manage TB we think it is a high time for the government to start implementing the Strategic and Action Plan for the Prevention and Control of Non-Communicable Diseases in Tanzania.

The current study has several limitations. First, since the study design is cross-sectional, it is difficult to tell changes which might have occurred over time regarding the readiness of diabetes facilities to manage TB. Therefore, the results should be interpreted with caution when comparing with other study designs. Also, the current study did not assess the availability of other important diagnostic methods for TB such as culture and molecular diagnostic techniques (Nucleic Acid Amplification Test and Whole Genome Sequencing). This is due to the fact that the letter is very sophisticated, costly, and rarely available in the peripheral healthcare facilities.

This study has a number of strengths. First, it is the first study to shed a light on the extent of availability and readiness of the diabetes facilities in Tanzania to provide TB management services. Second, the sample size was nationally representative hence the findings reflect the actual situation regarding the level of readiness to integrate DM and TB services in Tanzanian healthcare facilities.

## Conclusions

Most of the DM facilities had low availability and readiness to manage TB. The findings of our study display an urgent need to mobilize important resources to enhance the integration of TB services in DM facilities. This includes medications, management guidelines, diagnostics, and health professionals who have received refresher training on TB/DM co-management. However, presently, few DM facilities may be allowed to start managing TB as per the Strategic and Action Plan for the Prevention and Control of Non-Communicable Diseases in Tanzania 2016–2020.

## Data Availability

We received the administrative permission to access and use the dataset from the the ICF International, Rockville, Maryland, USA, through DHS program: the dataset is available on the repository https://dhsprogram.com/data/available-datasets.cfm

## Abbreviations

CD: Communicable Diseases
DHS: Demographic Health Survey
DM: Diabetes mellitus
HIV: Human Immunodeficiency Virus
ICF: Inner City Fund
LMICs: Low- and Middle-Income Countries
MoHCDEC: Ministry of Health, Community Development, Gender, Elderly and Children
NCDs: Non-Communicable Diseases
NTLP: National Tuberculosis and Leprosy Programme
SARA: Service Availability and Readiness Assessment
SDG: Sustainable Development Goals
TB: Tuberculosis
TB-DM: Tuberculosis-Diabetes
TSPA: Tanzania Service Provision Assessment
UNAIDS: United Nations Programme on HIV/AIDS
WHO: World Health Organization
